# The Combination of Abscisic Acid (ABA) and Water Stress Regulates the Epicuticular Wax Metabolism and Cuticle Properties of Detached Citrus Fruit

**DOI:** 10.3390/ijms221910242

**Published:** 2021-09-23

**Authors:** Paco Romero, María Teresa Lafuente

**Affiliations:** Department of Food Biotechnology, Institute of Chemistry and Food Technology (IATA-CSIC), Avenida Dr. Catedrático Agustín Escardino 7, 46980 Paterna, Valencia, Spain; mtlafuente@iata.csic.es

**Keywords:** ABA deficiency, fruit dehydration, gene expression, hormone application, Pinalate, postharvest

## Abstract

The phytohormone abscisic acid (ABA) is a major regulator of fruit response to water stress, and may influence cuticle properties and wax layer composition during fruit ripening. This study investigates the effects of ABA on epicuticular wax metabolism regulation in a citrus fruit cultivar with low ABA levels, called Pinalate (*Citrus sinensis* L. Osbeck), and how this relationship is influenced by water stress after detachment. Harvested ABA-treated fruit were exposed to water stress by storing them at low (30–35%) relative humidity. The total epicuticular wax load rose after fruit detachment, which ABA application decreased earlier and more markedly during fruit-dehydrating storage. ABA treatment changed the abundance of the separated wax fractions and the contents of most individual components, which reveals dependence on the exposure to postharvest water stress and different trends depending on storage duration. A correlation analysis supported these responses, which mostly fitted the expression patterns of the key genes involved in wax biosynthesis and transport. A cluster analysis indicated that storage duration is an important factor for the exogenous ABA influence and the postharvest environment on epicuticular wax composition, cuticle properties and fruit physiology. Dynamic ABA-mediated reconfiguration of wax metabolism is influenced by fruit exposure to water stress conditions.

## 1. Introduction

Abscisic acid (ABA) was first known as abscisin II [1,2] because it was identified as a substance that regulated cotton fruit and leaf abscission. Later studies revealed its role in both biotic and abiotic stresses, and highlighted the involvement of this hormone in the regulation of the molecular mechanisms underlying the response to dehydrating conditions in plants [3]. In fruit, the participation of ABA in fruit water stress regulation has also been demonstrated in both climacteric and non-climacteric fruit [4]. More recently, a relationship between ABA and cuticle, the first barrier to limit water loss, has been suggested in model plants [5,6] and during fruit ripening [7,8,9,10]. ABA treatment increased wax load in Arabidopsis plants, mainly due to changes in the alkane fraction [5]. Also, ABA deficiency increased cuticle permeability and resulted in thinner cuticles during tomato leaf development [6,9]. In contrast, deficient ABA levels in tomato fruit do not have a marked effect on wax content or composition, while ABA spraying increased wax load and cuticle thickness in cherry fruit [10]. In addition, a relationship between ABA and cuticle biology has been proposed in citrus fruit [7,8], although the putative ABA-mediated regulation of the cuticle composition and properties in fruit exposed to abiotic postharvest stresses remains elusive and needs to be further investigated.

The cuticle layer covers aerial plant parts and acts as the first barrier for the interaction with the environment. Given its lipophilic nature, it is a major water retention determinant in different plant organs, and is also involved in the regulation of temperature fluctuations and gas diffusion in addition to protection from pathogen invasion, among others [11,12,13]. Cuticular waxes are mixtures of cyclic compounds (e.g., triterpenoids) and aliphatic compounds (e.g., alkanes, fatty acids (FA), alcohols and aldehydes) derived from very-long-chain fatty acids (VLCFA) [14]. They can be divided into intracuticular and epicuticular waxes depending on whether their deposition is embedded in or on the cutin scaffold, respectively. Previous reports point out that fruit cuticles, and more specifically the epicuticular wax layer composition, is associated with fruit quality maintenance after harvest, in regard to susceptibility to water loss, pathogen infection, loss of firmness and the development of peel disorders [15,16,17,18,19,20,21].

Fruit cuticle composition is dependent on the species, and even on the cultivar, and is also determined by internal factors such as fruit development and ripening [7,8,9,22,23,24,25,26,27,28,29,30,31,32,33,34]. The effect of plant regulators, such as gibberellic acid, 2,4-dichlorophenoxy acetic acid, ethylene and 1-MCP, on epicuticular wax composition has been investigated [35,36,37,38,39], and a role for ABA in modulating the epicuticular wax metabolism and cuticle properties of fruit has been proposed [7,8,10]. Fruit cuticle composition and properties are also sensitive to external environmental factors such as humidity, temperature and light radiation [16,40,41]. Of these, water stress during the cultivation of or after harvesting fruit is considered the major factor that limits plant productivity and fruit quality after detachment, respectively. Cutin and intracuticular waxes have frequently been considered the main cuticle components responsible for reducing water permeability. Lately, however, the number of research works that correlate epicuticular wax load and composition with fruit water loss has grown [7,8,18,41,42,43]. Conversely, reports on how environmental humidity affects fruit cuticular wax metabolism are limited to tomatoes, grapes and pears [43,44,45], and whether ABA influences fruit cuticle properties as well as epicuticular wax content and composition during postharvest water stress remains elusive.

Citrus fruit is an important crop grown all over the world. As in most non-climacteric fruits, ABA plays a key role in citrus fruit development and ripening [46,47,48,49,50,51,52], but also in response to water stress [53,54,55,56,57,58]. Indeed, several studies have highlighted the relevance of endogenous ABA levels and an operational ABA signaling network in the molecular response of citrus fruit to dehydrating conditions determining their external fruit quality [53,55,59]. The availability of an impaired ABA biosynthesis mutant named Pinalate (*Citrus sinensis* L. Osbeck) has been crucial for such scientific progress to be made. A Pinalate orange is not a knockout mutant, but a spontaneous bud mutation that presents severe fruit-specific blockage of the carotenoid biosynthetic pathway, which results in yellow coloration, high susceptibility to postharvest dehydration and drastically low ABA levels in the flavedo (the outer colored peel part) [53,60,61,62]. It has also been reported that the epicuticular wax composition of this fruit differs from that in other orange cultivars with higher ABA levels during fruit ripening, despite the total wax load not being affected by low ABA content in the fully mature stage [8]. These findings converge with those in other citrus cultivars, pointing to a role for ABA in the regulation of cuticle biology during citrus fruit maturation [7]. However, no ABA feeding experiments have been performed and the relationship between ABA and citrus cuticles remains elusive. In addition, the effects of ABA or water stress on citrus cuticles after harvesting the fruit have never been investigated. By making the most of the markedly reduced ABA levels in Pinalate fruit, this study investigated whether ABA treatment drives changes in fruit cuticle properties as well as epicuticular wax load and composition after detachment, and if these modifications are influenced by exposure to water stress conditions during postharvest storage. We addressed these questions by comparing the wax composition as well as cuticle permeability and thickness, together with the fruit weight loss and firmness measurements, of the Pinalate fruit, either treated or not with ABA and left under high relative humidity (RH) or postharvest water stress conditions that favor fruit dehydration. Correlation analyses allowed us to identify the relations among ABA content, wax constituents, cuticle properties and fruit quality maintenance during storage. To dig more further into the molecular mechanisms underlying these relations, an expression analysis of the key genes involved in the biosynthesis and transport of wax components was performed.

## 2. Results

### 2.1. Effects of ABA and Water Stress on Wax Content and Wax Fraction Distribution

**Figure 1:**
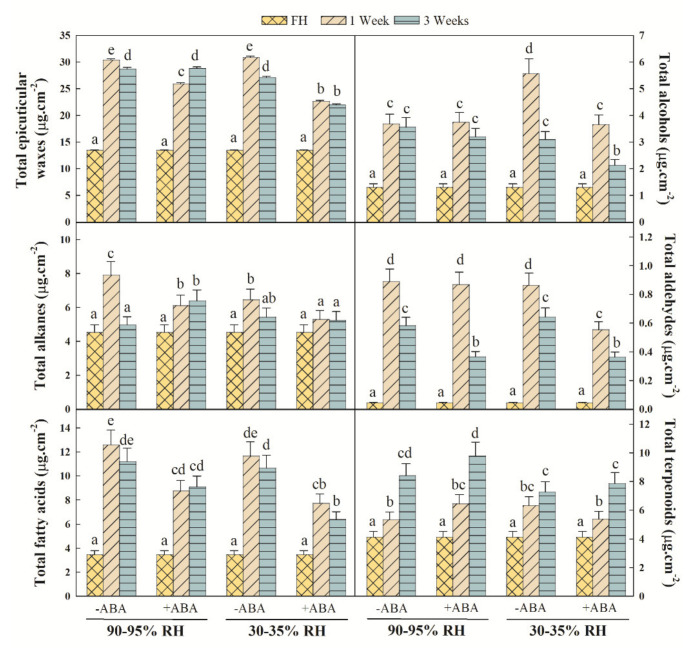
Effect of ABA and water stress on the total epicuticular wax and individual fractions content in Pinalate fruit. The effect of ABA (1 mM) treatment was evaluated together with the influence of high (90–95%) or low (30–35%) RH conditions on the total content (µg.cm^−2^) of epicuticular wax and the individual fractions up to 3 weeks of leaving Pinalate fruit at 20 °C. Bars are means ±SD of four replicates per condition. FH: Freshly harvested fruit. For each panel, the different letters above the bars indicate significant (*p* < 0.05) differences among conditions according to an ANOVA analysis followed by a Tukey test (*p* < 0.05).

Pinalate oranges were treated, or not, with 1 mM ABA solution before being stored under the control (90–95% RH) or water stress (30–35% RH) environmental conditions. As shown in Figure 1, the total wax content in Pinalate increased by two-fold in the first week of storage and slightly decreased thereafter regardless of the environmental conditions. ABA treatment reduced the total wax load increment observed by 1 week, which was more marked under the dehydrating conditions. By 3 weeks, this trend was observed only in the fruit stored under the postharvest water stress conditions (Figure 1).

Noticeable responses to the total content of five major wax fractions were detected in response to ABA treatment and the postharvest water stress conditions. These fractions were alkanes (C_22_-C_34_), FA (C_12_-C_28_), alcohols (C_22_-C_32_), aldehydes (C_24_, C_26_) and terpenoids (C_15_, C_29_ and C_30_) (Figure 1). The total alkane content increased by 1 week during the control or dehydrating fruit storage. Independently of the water stress environment, ABA treatment reduced this increase. Similarly, the increase observed in the total FA content after harvest was reduced by applying ABA to Pinalate fruit, regardless of storage conditions. In contrast, an effect of ABA on alcohol content was only observed in water-stressed fruit. Thus, the alcohol accumulation pattern in the Pinalate fruit under water stress remained after the ABA treatment, but the total achieved content lowered by about 30%. Aldehyde content increased by about nine-fold 1 week after fruit detachment and decreased thereafter during storage under both the control and water stress conditions. A major effect of ABA on aldehyde content was observed in the Pinalate fruit exposed to postharvest water stress, as this fraction accumulation lowered by about 25% and 30% by week one and week three, respectively, compared to untreated fruit. The total terpenoid content also increased after harvesting the fruit. No differences in terpenoid accumulation were observed between the ABA-treated and untreated fruit stored at either high or low RH, but the total content achieved in the ABA-treated fruit was higher at high RH than during water stress storage.

**Figure 2:**
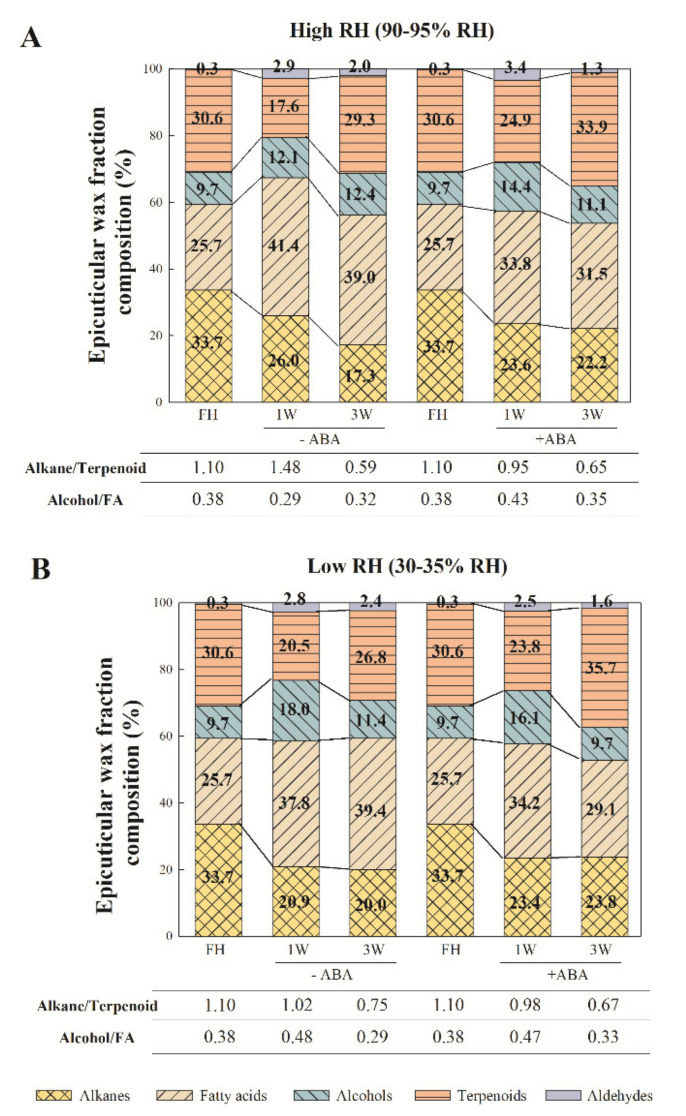
Effect of ABA and water stress on the proportion of epicuticular wax fractions in Pinalate fruit. The effect of ABA (1 mM) treatment on the percentage (%) of epicuticular wax fractions by 1 and 3 weeks (W) of Pinalate fruit storage at 20 °C was evaluated together with the influence of the (**A**) high (90–95%) or (**B**) low (30–35%) RH conditions. FH: Freshly harvested fruit. The values on the bars indicate the percentage of each fraction. The alkane/terpenoid and alcohol/FA ratios are indicated per condition. The same legend is used for panels (**A**,**B**).

The effects of ABA treatment on wax composition were dependent on exposure to postharvest water stress and storage duration (Figure 2). At harvest, the most abundant components were alkanes (33.7%), followed by terpenoids (30.6%), FA (25.7%), alcohols (9.7%) and aldehydes (0.3%). In response to harvest, alkane abundance lowered, and a transitory decrease in terpenoids was observed by 1 week. The percentage of FA and aldehydes increased 1 week after storage and remained almost steady thereafter under either environmental condition (Figure 2). Nevertheless, by 1 week of postharvest water stress, the abundance of alkanes and FA was lower, and that of alcohols and terpenoids was higher than in the fruit left at high RH. By 3 weeks, however, these trends were inversed and postharvest water stress brought about a decrease in terpenoid abundance and an increase in the alkane proportion. Hormone application enhanced the initial decrease in alkane abundance in the Pinalate fruit stored at high RH, while the opposite was found under the fruit-dehydrating condition. ABA treatment slightly modified the alcohol proportion by 1 week under either the control or postharvest water stress storage. The increase in the FA proportion after fruit detachment was attenuated by ABA application, and was more marked by 3 weeks under the fruit-dehydrating condition. Terpenoid abundance was increased by ABA independently of storage and period. However, the effects of ABA on the aldehyde proportion varied depending on postharvest water stress exposure and duration (Figure 2).

As a result of these changes, the alkane/terpenoid and alcohol/FA ratios, which are inversely related to fruit water retention, were modified in response to ABA application and/or fruit exposure to postharvest water stress. The alkane/terpenoid ratio transitorily increased 1 week after fruit detachment when stored at high RH, but continuously decreased in response to ABA treatment under this condition. In contrast, this ratio continuously lowered in the fruit exposed to postharvest water stress, and no influence of ABA was observed. The alcohol/FA ratio slightly varied during fruit storage. The effects of ABA were also minor. If any, an increase in the alcohol/FA ratio was observed in the ABA-treated fruit stored for 1 week at high RH compared to the untreated fruit (Figure 2).

### 2.2. Epicuticular Wax Composition Analysis

**Figure 3:**
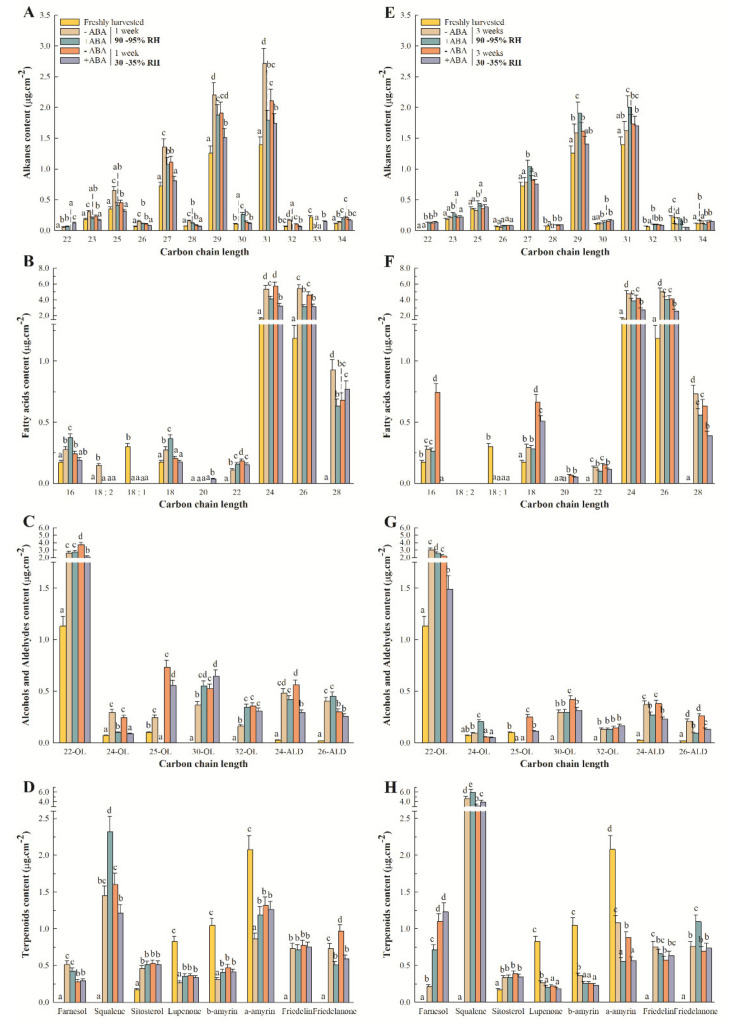
Effect of ABA and water stress on epicuticular wax constituents during Pinalate fruit storage. The effects of ABA (1 mM) treatment on the content of the epicuticular wax components were evaluated together with the influence of high (90–95%) or low (30–35%) RH by 1 (**A**–**D**) and 3 (**E**–**H**) weeks at 20 °C. (**A**,**E**) Alkanes; (**B**,**F**) fatty acids; (**C**,**G**) alcohols and aldehydes; and (**D**,**H**) terpenoids. Bars are the means ±SD of four replicates per condition. Different letters above the bars indicate significant (*p* < 0.05) differences among conditions according to an ANOVA analysis followed by a Tukey test (*p* < 0.05) for each component and storage time separately.

Heptacosane (C_27_), nonacosane (C_29_) and hentriacontane (C_31_) were the most abundant long-chain alkanes in Pinalate fruit, irrespective of ABA treatment and storage conditions (Figure 3A,E). The accumulation of tricosane (C_23_) and pentacosane (C_25_) was also noticeable among the odd-chain alkanes. Even-chain-length compounds (C_22_-C_34_) were a minority among alkane components and were not consistently detected under the assayed experimental conditions. The accumulation profiles of the odd long-chain alkanes were differently affected by ABA treatment depending on exposure or not to postharvest water stress. Some differences in their accumulation patterns were found when comparing high RH and fruit-dehydrating conditions. Thus, ABA treatment lowered the increments detected for the odd long-chain-length compounds by 1 week at high and low RH. In contrast, hormone application increased these compounds by 3 storage weeks at high RH, but did not modify their contents in the water-stressed fruit during this period. Their accumulation was higher at high rather than low RH by 1 week, but no differences caused by environmental conditions were found after 3 storage weeks in the untreated fruit (Figure 3A,E).

At harvest, the most abundant FA were lignoceric (C_24_) and cerotic (C_26_) acids, followed by palmitic (C_16_) and stearic (C_18_) acids. All these compounds, as well as montanic acid (C_28_), increased after harvesting fruit, but specific accumulation patterns were found in response to ABA application in addition to the storage period and conditions. Thus, the content of both lignoceric and cerotic acids increased during storage, and ABA treatment attenuated their accumulation independently of the environment (Figure 3B,F). In contrast, palmitic and stearic acids increased in response to ABA application by 1 week, but only at high RH. This effect was lost by 3 weeks. No effects of ABA on these FA were detected under the fruit-dehydrating condition by 1 week, but hormone treatment reduced their accumulation by 3 weeks. Similarly, exogenous ABA did not modify the montanic acid content under the water stress conditions, but its accumulation lowered at high RH by 1 and 3 weeks. In addition, hormone treatment reduced montanic acid accumulation under the fruit-dehydrating conditions by 3 weeks (Figure 3F).

The most abundant primary alcohols were docosanol (C_22_), tetracosanol (C_24_) and pentacosanol (C_25_). Triacontanol (C_30_) and dotriacontanol (C_32_) were not detected at harvest, but accumulated during storage (Figure 3C,G). All these compounds increased after harvest independently of the environmental conditions, but the effect of ABA treatment on the content of each compound depended on RH and storage duration. Thus, docosanol content, the most abundant alcohol in Pinalate fruit, was not affected by ABA treatment at high RH, but lowered under water stress by 1 week. In contrast, hormone application decreased the docosanol content by 3 weeks regardless of the storage conditions. Our analyses identified two aldehydes, tetracosanal (C_24_) and hexacosanal (C_26_), which increased after detachment, but did not show any remarkable differences between storage conditions (Figure 3C,G). ABA decreased their accumulation by 3 weeks. This trend was evident in tetracosanal content by 1 week, but only under postharvest water stress.

Among terpenoids, only α- and β-amyrins, lupenone and sitosterol were detected at harvest, although farnesol, squalene, friedelin and friedelanone accumulated thereafter (Figure 3D,H). Lupenone, α- and β-amyrins decreased in Pinalate fruit in response to fruit detachment and independently of storage conditions. The other terpenoids, however, increased after harvest. The response to ABA application was diverse among these compounds and depended on RH and storage duration. Sitosterol, friedelin and farnesol contents did not vary after ABA treatment, except for farnesol content, which increased in the ABA-treated fruit exclusively when stored for 3 weeks at high RH. Squalene content, the most abundant terpenoid in Pinalate fruit, was inversely regulated by ABA application between the control and fruit-dehydrating conditions by 1 week. By 3 weeks, its content had considerably increased and ABA induced its accumulation regardless of the environmental conditions.

### 2.3. Variations in Cuticle Properties and Fruit ABA Content, Firmness and Weight Loss

**Figure 4:**
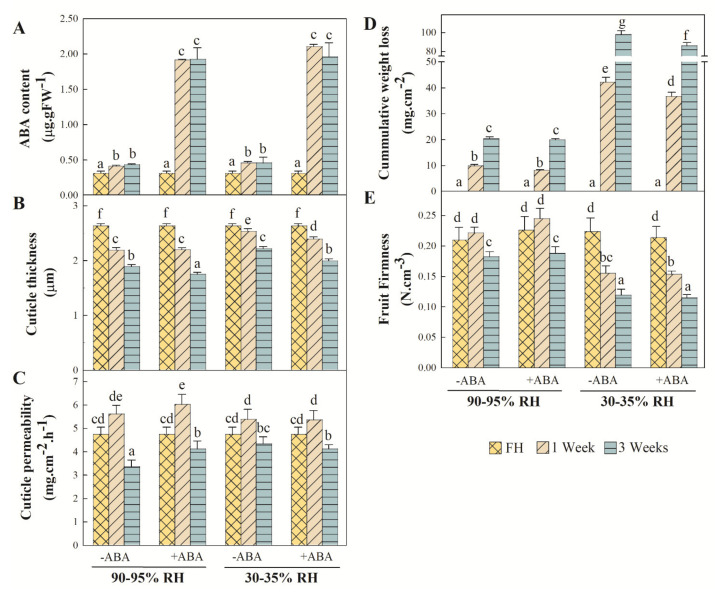
Effect of ABA and water stress on cuticle properties and fruit physiological parameters in Pinalate fruit. (**A**) ABA content is expressed as g per g of fresh weight (FW) of the flavedo. Bars are the means ±SD of three replicates of five fruit each. (**B**) Cuticle thickness. Bars represent the means ±SD of about 50 measurements for all three biological replicates analyzed per condition. (**C**) Cuticle permeability. Bars are the means ±SD of three replicates per condition. (**D**) Cumulative weight loss of Pinalate fruit calculated as fruit weight loss per surface area. Bars are the means ±SD of three replicates of 10 fruit each. (**E**) Fruit firmness was determined according to the intact fruit compression resistance load (N) and normalized by fruit size (cm^3^). Bars indicate the means ±SD of three replicates of 10 fruit each. For each studied parameter, different letters indicate the statistical (*p* < 0.05) differences in all the conditions together according to an ANOVA analysis followed by a Tukey test (*p* < 0.05).

The ABA content in Pinalate fruit slightly increased after detachment irrespective of storage conditions. Likewise, exogenous ABA application increased the hormone content in both the fruit stored at high RH and under postharvest water stress by about five-fold (Figure 4A). Cuticle thickness continuously decreased after detachment. By 1 week, this decrease was slight under postharvest water stress, but more drastic at high RH (Figure 4B). When the experiment ended (3 weeks), cuticles were thicker in the fruit left under the postharvest dehydration conditions than those left at high RH. ABA treatment did not significantly affect these patterns independently of storage. Nevertheless, the thinnest cuticles were those from the ABA-treated fruit stored for 3 weeks at high RH. Cuticle permeability (estimated as the cuticle transpiration rate) barely changed in response to the dehydrating environment and/or ABA treatment. In fact, an increase in cuticle permeability was detected only in the ABA-treated fruit stored for 3 weeks at high RH (Figure 4C). Cumulative weight loss per surface area continuously increased in Pinalate fruit during storage, and it was about four-fold higher when fruit were kept under the postharvest dehydration conditions (Figure 4D). A slight, but statistically significant, reduction in cumulative weight loss was observed by 1 and 3 weeks in the ABA-treated fruit versus the untreated fruit left under water stress. Fruit firmness remained unchanged by 1 week after detachment and decreased thereafter in the fruit left at high RH, and no effect of ABA was observed. Postharvest water stress, however, brought about a marked decrease in fruit firmness regardless of ABA treatment (Figure 4E).

### 2.4. Relationships between ABA Content, Cuticle Composition and Properties and Fruit Physiology 

**Figure 5:**
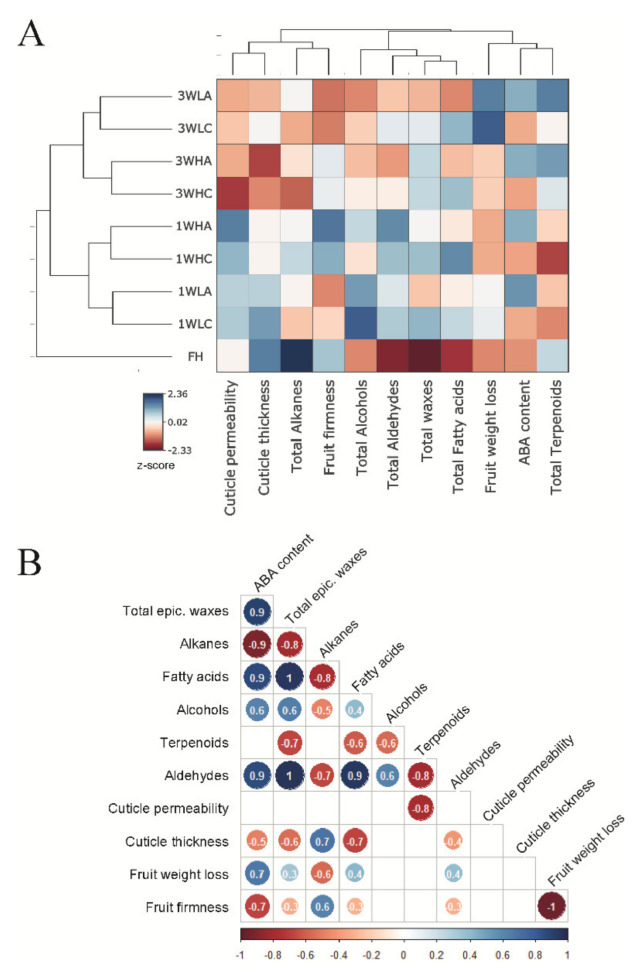
Clustering and correlation analyses of epicuticular wax composition, ABA content, cuticle properties and fruit physiological parameters in Pinalate fruit. (**A**) Hierarchical clustering analysis of the Pinalate fruit left at high (H, 90–95%) or low (L, 30–35%) RH for 1 week or 3 weeks (W), and treated (A, ABA) or not (C, control) with 1mM ABA, based on the chemical composition of their epicuticular wax layer, ABA content, cuticular properties and fruit physiology. The colors in the heatmap indicate the z-score value for each parameter and condition according to the scale in the legend. (**B**) Correlation matrix among the abundance of individual epicuticular wax fractions, ABA content, cuticle properties and fruit physiological parameters in the Pinalate fruit left under the above-described conditions. Numbers indicate the regression coefficient value, and only the statistically significant ones are colored according to the scale in the legend.

In order to elucidate the relationships among the experimental conditions as regards ABA application and exposure to postharvest water stress, the total epicuticular wax content, the content of each wax fraction, ABA levels, cuticle thickness and permeability, and fruit weight loss and firmness were used to perform a cluster analysis (Figure 5A). According to the HCA, the freshly harvested (FH) and stored fruit were the most clearly discriminated groups. Later, samples were clustered in a storage-period-dependent manner. During each storage period, the samples that were grouped depending on exposure to postharvest water stress followed a distinction between the ABA-treated and untreated fruit. The dendrogram corresponding to cuticle composition and properties, ABA content and fruit physiological parameters can be divided into three major groups. The first includes two subclades composed of cuticle thickness, permeability and fruit firmness in addition to total alkane content. Second, ABA content, total terpenoids and fruit weight loss were clustered together. The last group contained the total wax load and the total content of all the cuticle fractions, except alkanes and terpenoids (Figure 5A).

The effects of ABA application and exposure to water stress after fruit detachment on cuticle composition and properties, as well as on fruit weight loss and firmness, were further studied by a statistical correlation analysis (Figure 5B). ABA content correlated positively with total epicuticular wax (r = 0.9), FA (r = 0.9), alcohol (r = 0.6) and aldehyde (r = 0.9) proportions and fruit weight loss (r = 0.7). Inversely, ABA content negatively correlated with alkane proportion (r = 0.9), cuticle thickness (r = −0.5) and fruit firmness (r = −0.7). The alkane proportion showed a negative correlation with fruit weight loss (r = −0.6), but correlated positively with cuticle thickness (r = 0.7) and fruit firmness (r = 0.6). Inversely, FA and aldehyde abundance related positively to fruit weight loss (r = 0.4), but negatively to fruit firmness (r = −0.3) and cuticle thickness (r = −0.7 and r = −0.4, respectively). The percentage of terpenoids was the only wax fraction to show a correlation with cuticle permeability (r = −0.8) (Figure 5B). These relationships agreed with the correlations found among the different epicuticular wax fractions as alkanes negatively correlated with FA and aldehydes (r = −0.8 and r = −0.7, respectively) and FA related positively to the aldehyde proportion (r = 0.9). In turn, fruit weight loss negatively related to fruit firmness (r = −0.97) (Figure 5B).

### 2.5. Effects of ABA and Water Stress on the Transcriptional Regulation of Epicuticular Wax-Related Genes

**Figure 6:**
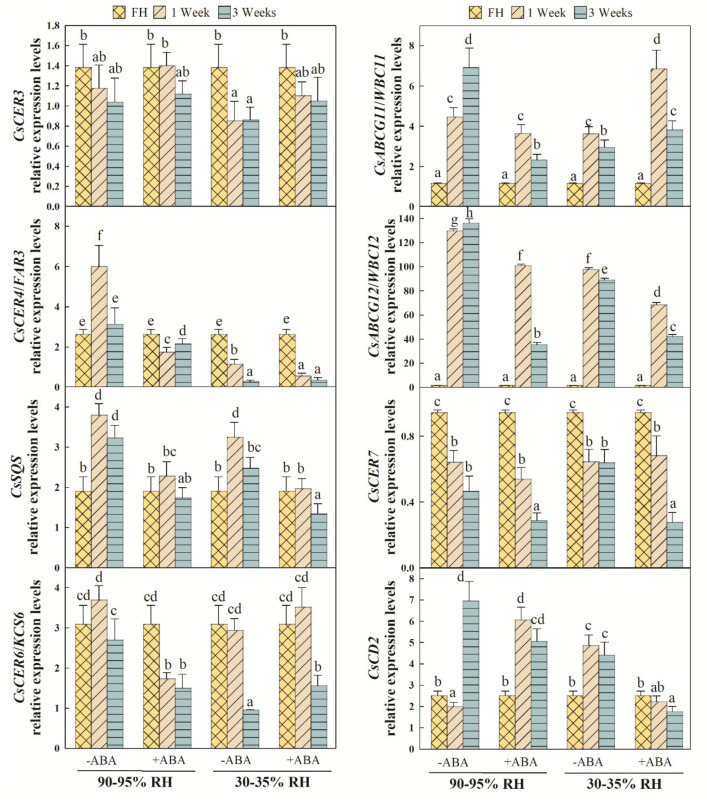
Effect of ABA and water stress on the transcriptional regulation of epicuticular wax metabolism. Relative expression levels of the genes related to the transcriptional (*CsCD2*) and post-transcriptional (*CsCER7*) regulation of the biosynthesis (*CsCER6/KCS6*, *CsCER3*, *CsCER4* and *CsSQS*) and transport (*CsABCG11/WBC11*, *CsABCG12/WBC12*) of the wax components in the Pinalate fruit treated (+ABA) or not (−ABA) with ABA (1 mM), and left at high (90–95%) or low (30–35%) RH for up to 3 weeks at 20 °C. Gene expression values are expressed as fold change levels of all conditions as compared to the freshly harvested (FH) fruit. Values are the means of three biological replicates per condition. Different letters indicate statistical (*p* < 0.05) differences among all the conditions together according to an ANOVA analysis followed by a Tukey test (*p* < 0.05) for each gene individually.

In order to further investigate a putative epicuticular wax metabolism reconfiguration mediated by ABA and/or exposure to postharvest dehydration, a transcriptional analysis on key genes involved in epicuticular wax biosynthesis, transport and regulation was performed (Figure 6). The expression of *CsCER3*, involved in the synthesis of alkanes, remained almost steady during fruit storage at high RH, but significantly decreased with exposure to fruit dehydration. The effect of ABA on *CsCER3* accumulation depended on the storage environment. Indeed, ABA treatment did not statistically affect *CsCER3* expression at high RH, but counteracted the decreased transcript accumulation pattern observed under water stress in the untreated fruit. The expression levels of *CsCER4/FAR3*, which encode an FA reductase involved in the synthesis of primary alcohols, transitorily peaked by 1 week at high RH, but continuously lowered when fruit were exposed to water stress after detachment. ABA treatment generally reduced the accumulation of transcripts regardless of storage conditions, although this effect was more marked at high RH. The gene expression levels of *CsSQS*, involved in squalene synthesis, increased after harvest, independently of storage, but this increment was transitory when fruit were left in a dehydrating environment. Independently of being exposed or not to postharvest water stress, ABA application reduced *CsSQS* transcript accumulation throughout the storage period. *CsCER6/KCS6,* which encode a β-ketoacyl-CoA synthase involved in the synthesis of VLCFA precursors, remained steady in the fruit left at high RH, but dropped by three-fold when fruit were exposed to dehydration for 3 weeks. The effects of ABA on the accumulation of *CsCER6/KCS6* transcripts differed depending on storage, because hormone treatment lowered the gene expression levels at high RH, but slightly increased the transcript levels by 3 weeks under postharvest water stress. Epicuticular wax transporters *CsABCG11/WBC11* and *CsABCG12/WBC12* were similarly regulated in regard to ABA and the water stress response. Both transporters were continuously induced during storage at high RH, and remained almost steady after an initial increase by 1 week when fruit were exposed to water stress. Transcript levels were also lower after water stress storage.

After ABA treatment, the *CsABCG11/WBC11* and *CsABCG12/WBC12* expression levels peaked by 1 week regardless of the water stress exposure. It is worth noting that ABA enhanced *CsABCG11/WBC11* accumulation, but lowered that of *CsABCG12/WBC12* under water stress compared to the untreated fruit. The expression levels of the *CsCER7* post-transcriptional regulator bottomed down in response to harvest by 1 week and remained steady thereafter independently of the environment. ABA also repressed transcript accumulation by 3 weeks at either high or low RH. Last, the *CsCD2* transcription factor expression showed a significant influence for both ABA and postharvest water stress exposure. Thus, *CsCD2* gene expression transiently decreased by 1 week to sharply increase thereafter at high RH, but increased in response to fruit dehydration in the first week of storage. In addition, ABA treatment counteracted the initial decrease in gene expression at high RH, while inhibiting the increment in the *CsCD2* transcript levels when fruit were exposed to water stress (Figure 6).

## 3. Discussion

The cuticle is extremely sensitive to surrounding fluctuations and, specifically, the epicuticular wax composition adjusts in response to environmental signals [63,64,65,66]. The study of the ABA-mediated regulation of cuticle properties and composition has been a hot research topic for years in both Arabidopsis model plants and several horticultural crops. However, research into this relationship in fruit has been limited to tomatoes, cherries and citruses [7,8,9,10]. In addition, whether ABA regulates epicuticular wax composition after fruit detachment or if such effects depend on fruit being exposed to dehydrating conditions remains elusive, despite ABA being the main hormone to regulate the fruit water stress response [4]. Moreover, cuticle properties influence fruit water loss and, hence, fruit quality during postharvest [11,15,16,40,66]. In citrus fruit, the association between epicuticular waxes and fruit water retention has been established [7,67]. There are also reports that moderated water stress (70–75% RH) during postharvest causes fruit dehydration and triggers ABA-mediated signals in order to reduce fruit water loss and, hence, alleviate external quality loss [53]. An important advance contributed by the present research is that the effect of increasing the fruit ABA content by hormone feeding on fruit epicuticular wax metabolism was studied in combination with exposure to water stress, which increased fruit weight loss after detachment. To this end, Pinalate, an ABA-biosynthesis-impaired citrus mutant cultivar, that displays sharply reduced hormone levels in the flavedo, was treated with ABA and exposed to postharvest water stress.

The major finding in the present work is that ABA treatment dynamically modifies the epicuticular wax composition and metabolism of Pinalate fruit after detachment depending on whether the fruit is exposed, or not, to postharvest dehydration, and also on the duration of stress. This is deduced from the results, which showed that the increased total epicuticular wax load observed after fruit detachment was diminished by ABA treatment only after 1 week at high RH, but more markedly and during complete storage (up to 3 weeks) under fruit water stress (Figure 1). These ABA application effects were also evident on the total contents of individual wax fractions, such as alkanes, alcohols and aldehydes, and best-fitted the accumulation profile of FA, but not terpenoids (Figure 1). The compositional data analysis of each wax fraction further supported our statement as different responses to ABA treatment were observed by 1 and 3 weeks, which depended on the storage conditions for most individual wax components (Figure 3). In this context, the effects of ABA treatment on modifying wax composition were more evident when focusing on the proportion of each separate fraction. The most important changes can be summarized as attenuated FA accumulation and the enhanced increase in the terpenoid proportion in the ABA-treated fruit compared to those untreated, regardless of the postharvest storage conditions (Figure 2). In fact, even though ABA content correlated with the total epicuticular wax load and to all the wax fraction abundances, except terpenoids (Figure 5B), the contents of several terpenoid compounds, such as squalene, lupenone, friedelanone and α- and β-amyrins, were regulated by ABA application (Figure 3D,H). However, this ABA-mediated regulation differed between high RH and water stress, and also between 1 and 3 storage weeks, which probably modified their abundance in this fraction, and might influence both cuticle permeability and fruit weight loss [36,68,69,70,71]. This agrees with the fact that ABA treatment increased cuticle permeability by 3 weeks at high RH, but reduced Pinalate fruit weight loss during postharvest dehydration (Figure 4C,D).

Currently, research into the effects of postharvest water stress on wax composition is limited to pear and tomato; Korla pear wax content and composition adjusts to different RH storage conditions, while the wax chemical profile of tomato fruit, including a long-shelf-life cultivar, does not modify in response to water stress after harvesting the fruit [43,45]. In the present study, orange fruit displaying sharply low ABA levels showed increased total wax load after fruit detachment, without influence from the water stress of the postharvest environment. On the contrary, the total contents of the alkanes and alcohols in Pinalate were influenced by storage conditions by 1 week (Figure 1). Furthermore, under the postharvest dehydration conditions, the abundance of alkanes and FA was lower, and that of alcohols and terpenoids was higher after this period. Moreover, by 3 weeks, these trends were reversed as postharvest water stress brought about a decrease in terpenoid abundance and an increase in alkane proportion. Consequently, the alkane/terpenoid and alcohol/FA ratios varied with both RH and storage duration (Figure 2). Several research works converge with the idea that the proportion of hydrocarbons (i.e., alkanes), unlike terpenoids and cyclic compounds, is the most effective fraction for water retention. Thus, a drop in the alkane/terpenoid ratio would increase the proportion of amorphous structures and, consequently, cuticle permeability and/or fruit water loss [36,68,69,70,71,72]. Results from our chemical analysis partially agree with these findings, as the alkane/terpenoid ratio dropped in response to water stress by 1 week when a four-fold increase in cumulative fruit weight loss was noted (Figure 2 and Figure 4). Notwithstanding, ABA application lowered the alkane/terpenoid ratio after 1 week of storage at high RH, while no differences in fruit weight loss or cuticle permeability were found between the ABA-treated and untreated fruit left under this condition. Conversely, fruit weight loss statistically decreased as a result of ABA treatment under postharvest dehydration, but the alkane/terpenoid ratio in these fruit barely changed compared to the untreated fruit (Figure 2 and Figure 4). Besides, a higher alcohol/FA ratio has been associated with greater fruit weight loss [45], which agrees with our results showing that the dehydrating condition increased the alcohol/FA ratio compared to remaining at a high RH for 1 week. Minor ABA effects on this ratio were observed. If any, ABA treatment increased the alcohol/FA ratio by about 50% by 1 week at high RH, which was due mainly to a reduction in FA abundance (Figure 2 and Figure 4). Altogether, the results presented herein partially diverge from the idea of a straight correlation between these ratios and the fruit cuticle permeability and susceptibility to weight loss. In addition, the attenuated total epicuticular wax accumulation as a result of ABA treatment after Pinalate fruit detachment contrasts with not only reported increments in wax content in cherry fruit after on-vine spraying with ABA [10], but also with the fact that ABA-deficient tomato fruit mutants do not display differences in total wax content compared to their parental lines [9]. The epicuticular wax load of the fully mature Pinalate fruit did not differ from that in other citrus cultivars with higher endogenous ABA contents [8]. In this context, we should bear in mind that Pinalate fruit presents low ABA levels in the peel, but it is not a knockout mutant, and ABA treatment applied to orange fruit with higher ABA levels has not led to changes in either susceptibility to fruit weight loss or the molecular mechanisms underlying fruit dehydration, probably because endogenous levels of the hormone might suffice to trigger cellular processes to cope with stress [53,60,62]. These facts indicate that the ABA-mediated regulation of cuticle metabolism is species-/cultivar-dependent, and other intrinsic factors, such as endogenous hormone levels, climacteric or non-climacteric fruit ripening and/or fruit sensitivity to hormone application when on-vine or detached, might influence this relationship.

The expression profiles of the key genes involved in the synthesis, transport and transcriptional regulation of epicuticular waxes generally support the notion that the effect of ABA on this metabolism depends on both RH and the duration of the imposed water stress after harvesting the fruit. Indeed, the expression patterns of all the studied genes were somewhat influenced by ABA. Despite the lack of statistical differences in the *CsCER3* levels between the ABA-treated and untreated fruit stored in either RH environment, it was noteworthy that ABA application alleviated the diminished transcript accumulation of this gene, which is involved in alkane synthesis during postharvest dehydration (Figure 6). However, the effect of ABA decreasing the expression of *CsSQS* and *CsWBC12*, both involved in the synthesis of squalene and terpenoid precursors and in transporting cuticular components to the extracellular matrix, respectively, was observed regardless of storage duration and conditions. This was also true for *CsCER7*, a post-transcriptional regulator, accumulation despite the effect of ABA decreasing gene expression depending on storage duration, since it was only observed by 3 weeks (Figure 6). In contrast, the effects of ABA on *CsCER4*, *CsCER6*, *CsWBC11* and *CsCD2* regulation were dependent on both exposure to postharvest dehydration and stress duration. Moreover, the expression of the *CsWBC11* transporter and the *CsCD2* transcription factor was affected by ABA via a trend that did not vary between 1 and 3 weeks when fruit were stored under the dehydrating condition, but it differed between the storage periods at high RH. Inversely, *CsCER6* and *CsCER4*, respectively involved in the synthesis of VLCA and primary alcohols, showed an ABA effect that did not depend on storage period at high RH, but varied between 1 and 3 weeks when fruit were exposed to water stress. Altogether these data reflect that the ABA-mediated transcriptional regulation of epicuticular wax metabolism is complex and depends on the ABA treatment and postharvest water stress combination, and this relation may change as stress exposure progresses.

Our results indicate that detachment from tree causes the accumulation of epicuticular wax load in citrus fruit with low ABA levels, and that applying ABA after harvesting the fruit attenuates this increase. This ABA-related effect substantially increases in fruit exposed to water stress during postharvest. In turn, this research reveals that ABA-driven changes in the epicuticular wax chemical profile and metabolism depend firstly on storage duration, and in a minor extent on the exposure to water stress conditions during postharvest. Therefore, this data provides clues for industrial wax synthesis purposes and improves the knowledge on the ABA-dependent regulation of cuticular wax metabolism in fruits.

## 4. Materials and Methods

### 4.1. Fruit Materials and Experimental Design

Pinalate (*Citrus sinensis* L. Osbeck) sweet orange fruit were harvested from five adult trees grown in experimental orchards in the fully mature ripening stage according to the normal cultural practices applied by the Citrus Germplasm Bank at the IVIA (Moncada, Valencia) in Spain. Fruit were immediately delivered to the laboratory and those without peel damages or visual defects and with an average diameter of 6 cm were assigned to two groups. Fruit were treated with either ABA by dipping them for 1 min in an aqueous 1 mM ABA solution containing 0.7% ethanol to dissolve the hormone (treatment group, +ABA) or water containing 0.7% ethanol (control group, −ABA) following the same procedure. ABA treatment was repeated 2 weeks after harvest to ensure high ABA levels throughout the experiment. This dosage was selected according to previous experiments performed by our group which showed the effect of exogenous ABA on the response of citrus fruit to stressful conditions [53,55,59]. Both sets of fruit were divided into two subgroups and stored in the dark at 20 °C for up to 3 weeks in incubation chambers under control (90–95% RH, subgroup 1) or dehydrating (30–35% RH, subgroup 2) conditions. Samples were collected at harvest (freshly harvested fruit, FH), and the ABA-treated and untreated fruit were left for 1 and 3 weeks in the control or dehydrating environments (9 different conditions). For all the conditions, four biological replicates, each consisting of five fruit, were used for the wax content and composition analyses. Three additional biological replicates of 10 fruit each per condition were used to take fruit firmness and weight loss measurements. For all the conditions, three biological replicates of five fruit each were employed to determine cuticle thickness and permeability, and to collect flavedo samples, which were frozen and homogenized in liquid nitrogen, and kept at −80 °C for the ABA and gene expression analyses. Therefore, all nine samples composing the experimental design consisted of 65 fruit each.

### 4.2. Cuticular Wax Analysis

Epicuticular waxes from intact fruit of known surface areas were extracted by dipping fruit for 1 min in two successive chloroform baths, the first of which contained 100 µg of tetracosane as the internal standard. Waxes were derivatized using BSTFA and resuspended in 100 µL of chloroform after the evaporation of excess BSTFA, as previously described [43]. Wax extracts were injected into a gas chromatograph (GC) 7890B system (Agilent) equipped with an HP-5MS UI (30 m × 250 µm × 0.25 µm) column (Agilent Technologies, Santa Clara, CA, USA) and a 5977A simple quadrupole detector (Agilent Technologies) at the SCSIE-UV Gas Chromatography Facility (Valencia, Spain) following the conditions in [8]. Briefly, the oven temperature was held at 70 °C for 2 min before being raised by 10 °C min^−1^ to 200 °C, by 3 °C min^−1^ to 300 °C and finally held for 20 min. The injector temperature was 250 °C. The comparison of the relative retention times with those of commercial standards was used to identify most of the wax components. Computer matching against commercial (Nist, wiley7n) libraries and by MS literature data was also used for identification.

### 4.3. Cuticle Permeability and Thickness

Peel disks of 3 cm diameter were excised from the equatorial zone of intact fruit. Cuticles were enzymatically isolated and their permeability was measured in gravimetric chambers as described in detail in [8]. Briefly, isolated cuticles were placed face-up in 3D-printed chambers which exposed a constant cuticle surface area, which acted as the only separation barrier between the known amount of water inside the chamber and the dehydrating environment of a desiccation container (25 °C, 0% RH). Folded chambers were stored for up to 1 week and weight loss was measured daily. Cuticle permeability was estimated as the weight loss per hour and per unit of cuticle surface area. At the end of the assay, the integrity of cuticles was verified by 0.01% aqueous solution (*w*/*v*) of Toluidine Blue O (Merck, Darmstadt, Germany) staining. One disk of all five fruit, composing a biological replicate per sample, was used to obtain the isolated cuticles, and three biological replicates per sample were analyzed. Cuticle thickness was determined by light microscopy. Pericarp cubes were excised from fruit, and tissue fixation and embedding were performed as in [9]. A solution of Oil Red O (Alfa Aesar, Kandel, Germany) in isopropyl alcohol was applied to 10 µm sections. The stained slides were visualized under an Eclipse 90i Nikon microscope (Nikon corporation, Tokyo, Japan) with a 40X objective and which used the Nis Elements BR 3.2 software (Nikon corporation, Japan). The distance between the outer cuticle part and the top of the most external epidermal cell was used to measure cuticle thickness by the Fiji software (ImageJ 1.49q Software, National Institutes of Health, Bethesda, MD, USA), as previously described by [8]. One pericarp cube from the equatorial zone of all five fruit, composing a biological replicate per sample, was excised, and three biological replicates per sample were analyzed. About five measurements were taken in each section (roughly 75 values per sample).

### 4.4. ABA Analysis

As previously described [51], ABA was extracted from 1 g of fresh weight frozen flavedo with 80% acetone, containing 0.5 g L^−1^ of citric acid and 100 mg L^−1^ of butylated hydroxytoluene. After centrifuging, the supernatant was three-serial diluted in ice-cold TBS (6.05 g Tris, 8.8 g L^−1^ of NaCl and 0.2 mg L^−1^ of Mg Cl_2_, pH 7.8) and three samples for each dilution were analyzed by indirect ELISA.

### 4.5. Fruit Weight Loss and Firmness Determinations

During storage, Pinalate fruit were weighed daily to determine the amount of water loss. Fruit cumulative weight loss was calculated as the amount of water loss per surface area. Fruit surface area was determined by measuring three diameters per fruit for every biological replicate. Fruit firmness was analyzed as in [73] with minor modifications. Fruit compression resistance, based on a 5 mm deformation at two points of the fruit equator, was measured by a 4502 Instron Testing Machine (Instron).

### 4.6. Clustering Analysis

A hierarchical cluster analysis (HCA) was performed to group the different conditions in the experimental design according to their similarities in terms of both cuticle composition and properties in addition to the measured fruit physiological parameters. Average linkage clustering and Euclidian distance methods were followed to plot the dendrogram and heatmap (www.heatmapper.ca (accessed on 5 April 2021)).

### 4.7. RNA Extraction, cDNA Synthesis and RT-qPCR

RNA extraction, cDNA synthesis and RT-qPCR were performed following previously described well-established protocols [50]. Total RNA was isolated from flavedo samples, and 2 µg were used for the first-strand cDNA synthesis with the “Maxima H Minus First Strand cDNA Synthesis kit with dsDNase” (Thermo Scientific). The specific primer pairs for the genes of interest (*CsCER3*, *CsCER4*/*FAR3*, *CsCER6*/*KCS6*, *CsSQS*, *CsABCG11*/*WBC11*, *CsABCG12*/*WBC12*, *CsCD2* and *CsCER7*) and those employed for data normalization (*CsACT* and *CsTUB*) (Supplementary Materials Table S1) were mixed with SYBR Green to monitor cDNA amplification in a LyghCycler480 System (Roche Diagnostic). Amplicon specificity was determined by a melting curve analysis. Fold change relative gene expression values of the target genes were obtained by the Relative Expression Software Tool (REST, rest.gene-quantification.info), as previously described in [50]. Three independent biological replicates and two technical replicates were performed per sample.

### 4.8. Statistical Analyses

Statistical analyses were performed using the INFOSTAT software. Data of the parametric variables were subjected to an analysis of variance (ANOVA), and the significance of differences was determined by Tukey’s test (*p* < 0.05) on the mean values. Correlation analyses, carried out with the R software, established the relations among wax fractions and components, cuticle properties and fruit quality parameters. The statistical significance of the positive and negative correlations was considered at *p* < 0.05.

## Supplementary Materials

The following are available online at https://www.mdpi.com/article/10.3390/ijms221910242/s1, Table S1: Primers used for qPCR analysis.
