# Supplementary material for: The Combination of Abscisic Acid (ABA) and Water Stress Regulates the Epicuticular Wax Metabolism and Cuticle Properties of Detached Citrus Fruit
Source: Int J Mol Sci. 2021 Sep 23;22(19):10242. doi: 10.3390/ijms221910242 (PMC8549707; doi:10.3390/ijms221910242)
Supplement: Supplementary file 1 [file ijms-22-10242-s001.zip › ijms-1332114-supplementary.pdf]

Table S1. Primers used for gene expression analyses.

Citrus ID	Short name	Forward Sequence (5' - 3')	Reverse Sequence (5' - 3')
orange1.1g024218m	*CsCD2*	GGCGTATTGCCAACATTCTT	CCTCACGCTCAAAAGTCTCC
orange1.1g043337m	*CsCER7*	GGTCGCAACCCTTTTGAATA	GGATCAGCCATTGGAGAGAA
orange1.1g006768m	*CsCER3*	CGGGGAGTCGATTTCAAGCA	TAATGTTGCATGCCCAGCTG
orange1.1g008315m	*CsCER4/FAR3*	GACAACAAAAGGCAGTCGGAC	ACAAATTGGTGCTGGGCCTA
orange1.1g015087m	*CsSQS*	AGTCAAAATGAGGCGTGGACT	TACTTGCTTGCTCGGGTGG
orange1.1g011007m	*CsCER6/KCS6*	ACTTCATGTCCAAGCCAAGG	GCAGGTCTCTTCACCGAGTC
orange1.1g005142m	*CsWBC11*	GATTTCTACCAAACTTCTCAGCACTCG	ATTGTAACCAGTTCCCACGTTCAGAT
orange1.1g046969m	*CsWBC12*	AGCAAAGTTGGCTGTGGAGT	TCTCAGCTGTTGCCATGTTC
orange1.1g013062m	*CsACT*	TTAACCCCAAGGCCAACAGA	TCCCTCATAGATTGGTACAGTATGAGA
orange1.1g013062m	*CsTUB*	GCATCTTGAACCCGGTAC	ATCAATTCGGCGCCTTCAG
